# Oxidative stress and the unfulfilled promises of antioxidant agents

**DOI:** 10.3332/ecancer.2015.556

**Published:** 2015-07-23

**Authors:** Marco Giorgio

**Affiliations:** Department of Experimental Oncology, Institute of Oncology, Via Adamello 16, 20139, Milan, Italy

**Keywords:** oxidative stress, reactive oxygen species, mitochondrial respiration, redox signalling, antioxidant, cancer

## Abstract

It is well known that aging and its associated diseases, including cancer, are triggered by oxidative damage to biological macromolecules. However, antioxidant compounds are still disappointingly distant from any clinical application, so that Jim Watson has declared that antioxidant supplementation may have caused more cancers than it has prevented Watson J ((2013) **Oxidants, antioxidants and the current incurability of metastatic cancers** *Open Biol* **3** DOI: 10.1098/rsob.120144).

To clarify this paradox, here, we describe the mechanisms of oxidative stress focusing in particular on redox balance and physiological oxidative signals.

## What is oxidative stress?

Oxidative stress indicates a condition occurring when oxidising substances accumulate and accidental oxidative reactions thrive. In mammals, such as in all aerobic eukaryotes, the molecules with substantial oxidising potential contain oxygen. In particular, both reactive oxygen species (ROS), including singlet O_2_, superoxide anion (O_2_^−^), hydrogen peroxide (H_2_O_2_), and hydroxyl radical (OH^−^), and reactive nitrogen species (RNS), including peroxynitrite (ONOO^−^), nitrogen dioxide (NO_2_), and dinitrogen trioxide (N_2_O_3_), are potent oxidising agents in living organisms [2].

In cells, the accumulation of ROS and, subsequently, RNS increases the chance of formation of oxidative modifications in proteins, resulting in protein carbonyl content and oxidised- or nitro-modified residues, in lipids, generating hydroperoxide lipid derivatives, and in both purines and pyrimidines, inducing DNA adducts and breaks. Ultimately, the rise of oxidative attacks to biological macromolecules leads to the dysfunctions of proteins, membranes, and nucleic acids.

## How do antioxidants work?

Antioxidants are substances that can neutralise ROS and RNS by accepting or donating electrons and, as a consequence, might be converted into a radical form containing unpaired electrons that are less reactive than the neutralised ROS or RNS. Then, another antioxidant molecule may regenerate the paired form of the radical antioxidant. Loosely held hydrogen, large aromatic structures, and unsaturated bonds are all potent electron acceptor sites able to neutralise the free radicals present in antioxidant molecules [3]. Several plant metabolites contain these antioxidant moieties, for example, the large class of polyphenol compounds, including quercitin, myricetin, catechins, anthocyanins, that are present in food sources [4].

Together with these plant derivatives, a variety of different antioxidants are available for clinical purposes. These include endogenous molecules, such as α-tocopherol, β-carotene, glutathione, ascorbic acid, adenosine, lipoic acid, coenzyme Q, lactoferrin, as well as synthetic antioxidants, such as thiols, ebselen (selenium-based peroxide scavenger) [5], idebenone (coenzyme Q-analogue) and the more recently developed mitoQ (mitochondrial-targeted ubiquinone) [6], or α-tocopheryl-succinate nanoparticles [7]. Notably, several cardioprotective drugs, such as probucol and carvediol, or neuroprotective drugs, such as edaravone, are potent and effective antioxidant against the overload of ROS upon ischemia/reperfusion [8].

Other compounds, often included in the category of antioxidants, increase instead the endogenous levels of endogenous antioxidants such as *n*-acetyl cysteine (precursor of glutathione) or inhibit ROS production from cellular oxidases (allopurinol) or from metal ion reactions as in the case of iron chelators (deferoxamine) [3].

## Antioxidant failure in clinical studies

The present day is a golden age for antioxidants, promoted by the popularity of the free-radical theory of aging. Eating food rich in antioxidants protects from cancer and heart disease is common in particular.

Since the last three decades, the establishment of pre-clinical evidence showing that antioxidants protect deoxyribo nucleic acid (DNA) from being damaged by oxygen free radicals, potentially preventing the genetic mutations that cause cancer [9]. Antioxidants have also been consistently shown to reduce oxidative damage to low-density lipoprotein (LDL) cholesterol inside atherosclerotic plaques, thus protecting against atherosclerosis in the walls of arteries [10]. Thus, antioxidant treatments was promising to increase longevity by defeating the putative major cause of aging, that is, oxidative stress, or, more specifically by fighting top killers such as cancer and cardiovascular disease.

Unfortunately, several large randomised clinical trials found that antioxidant supplementation does not reduce the risk of cardiovascular disease or cancer, whereas one antioxidant, β-carotene, actually appears to increase the risk of some types of cancer in smokers. From 1985, the α-tocopherol/β-carotene cancer prevention trial (ATBC) was the first large study to examine the effect of antioxidants. Aged male smokers who had assumed 20 mg β-carotene for 8 years had an 18% increase in lung cancer incidence, and a less significant increase in prostate cancer, with respect to the placebo group [11]. In the following years, the beneficial effects of supplementation with β-carotene or vitamin A, α-tocopherol, ascorbic acid, and selenium increasingly became the object of debate [12–17].

Presently, after reviewing all the information, the US Preventive Services Task Force recommends against β-carotene or vitamin E to prevent cancer and cardiovascular disease (http://www.uspreventiveservicestaskforce.org) [18].

Furthermore, regardless of the fact that some epidemiological studies have overall shown that vegetable-enriched diets, while increasing antioxidant intake [19], inversely relate to mortality [20] or cancer [21] and stroke [22] risks, and that, in animal models, antioxidant-rich foods appears to inhibit tumorigenesis [23] and to be cardioprotective [24], both the US food and drug administration [25] and the European food safety authority [26] have banned any writing that could imply potential health benefits on the package labels of products with antioxidants.

Actually, the cancer prevention recommendations of eating, mostly, food of plant origin indicated by international health organisations such as the World Health Organisation (www.who.int/dietphysicalactivity/whatworks) or the World Cancer Research Fund International [27] are not related to vegetable antioxidant supply.

## Insight into ROS metabolism

The negative effect of oxygen on living organisms has been known for a long time [28]. However, although a negative correlation was observed in vertebrates between the intracellular levels of ROS/oxidative stress and longevity, the levels of endogenous antioxidants were also found to anti-correlate with life span. The supplementation of antioxidants in animal models, including frogs, pigs, rats and mice, did not affect mortality [29]. In actual fact, oxidative stress is determined by the rates of both ROS production and scavenging. Thus, the negative effect of aerobic metabolism could be only partially balanced by antioxidant activities if ROS production is maintained.

ROS are usually considered as a side effect of aerobic metabolism, and mitochondrial respiration is thought to be the main intracellular source of accidental ROS [30]. During mitochondrial respiration, electrons are extracted from nicotinamide adenine dinucleotide (NADH) or succinate and are then transferred to O_2_ through a chain of enzymatic complexes. In the final step of this electron-transfer chain (ETC), the cytochrome c oxidase (complex IV) catalyses the full reduction of molecular O_2_ to water, without forming O_2_ radicals. However, partial reduction of O_2_ leading to the formation of O_2_^−^ can occur if O_2_ hits sensible reduced sites of the ETC upstream of complex IV [31, 32]. Experimental data indicate that ROS are indeed continuously produced during mitochondrial respiration and that up to 2% of the total O_2_ consumption is converted to ROS [30].

Cells are normally able to defend themselves against ROS damage through the use of specific enzymatic (dismutases, catalase, peroxidases) or non-enzymatic (A, C, and E vitamins, uric acid, bilirubin) ROS-reducing mechanisms. The O_2_^−^ dismutase enzyme, for instance, catalyses the conversion of O_2_^−^ into H_2_O_2_, which is, in turn, reduced to water by the glutathione peroxidase and the catalase enzymes. In this way, the levels of different ROS are lowered to avoid the excessive oxidation of cellular components [30].

The rate of aging is assumed to be influenced, at least in part, by the rate of ROS production rather than by the rate of ROS scavenging. However, increased ROS production during aging is controversial; in contrast, scavenging activity has been clearly found to decrease over a lifetime and in different degenerative diseases [33–35].

## Oxidative stress has a physiological role

Substantial evidence demonstrates that ROS have a physiological role regardless of their toxicity. In fact, ROS have been shown to mediate growth factor/hormone/cytokine signal transduction, to regulate gene expression, and to determine programmed cell death [36, 37]. Among ROS, H_2_O_2_ is diffusible, less reactive and longer-lived than, for instance, O_2_^−^ and OH^−^. H_2_O_2_ is especially involved in the regulation of intracellular signalling pathways and could be considered as a second messenger [37].

Since the rate of mitochondrial ROS formation depends on the local concentrations of O_2_ and energetic substrates, and on ATP cellular demands [38, 39], the emerging picture is that mitochondria generate ROS in a regulated manner to deal with the different metabolic activities of the cell [40] and/or hypoxic conditions [41, 42].

ROS do not only cause irreversible damage to cellular components, they can also lead to fully reversible protein modifications. In particular, H_2_O_2_ has been demonstrated to directly oxidise cysteinyl thiols inducing formation of disulphide bonds and sulphenic acids. It has also been shown to induce glutathionylation of cysteine residues or the formation of methionine sulphoxide on methionine residues in a variety of contexts, such as the transcription factors OxyR and Pap1 in bacteria and in the yeast, respectively, the Kinase Sty1 in the yeast, the vacuolar ATPase, Vatp, in plants, the HIV-2 protease in viruses, the arylamine N-acetyl transferase 1, NAT1, the indoleamine 2, 3-dioxigenase, the phospholipase A2, iPLA2Beta, the small ubiquitin-related modifiers SUMO E1 subunit Uba2 and SUMO E2-conjugating enzymes Ubc9, the phosphatases PTP1B (protein tyrosine phosphatase 1B) and PTEN (phosphatase and tensin homologue), the peroxidase enzymes Prx I and II (both only cytosolic) and Prx III (cytosolic and mitochondrial), the annexin A2 protein, the heat shock factor 1 (HSF1) the mitochondrial enzymes aconitase and α-ketoglutarate dehydrogenase, and the subunits of the respiratory complex I, in mammals [43].

Overall, it has been demonstrated that oxidation and reduction of key cellular proteins participate in a redox-dependent regulation of cellular functions, including energy metabolism and response to stress. It has also become clear that intracellular signalling pathways can be activated by changes in intracellular metabolic redox reactions that involve O_2_^−^ and H_2_O_2_ [44]. Early hypotheses had proposed that exposure to specific environmental factors can induce ROS accumulation, thus triggering abnormal ROS-signalling leading to increase proliferation and malignant transformation. Evidence for this was obtained from studies showing that carcinogen initiators and promoters, including ionising radiation and polycyclic aromatic hydrocarbons, increase ROS formation, which in turn favours tumorigenesis [45]. A clear mechanism of how H_2_O_2_ in particular, can favour proliferation has emerged from studies on the redox regulation of critical phosphatases involved in signal transduction from plasma membrane receptors, together with the findings that several growth factors, such as EGF or Insulin/IGF, trigger H_2_O_2_ production directly from their membrane receptors [51, 79].

In this context, the function of the p66Shc protein is representative. P66Shc is the largest of the three isoforms encoded by the *ShcA* locus and almost ubiquitously expressed in vertebrates; it functions to regulate intracellular ROS levels and mitochondrial apoptosis. Cytosolic p66Shc mediates activation of the membrane oxidase activity and suppresses catalase and MnSOD expressions [46]. Then, a fraction of p66Shc translocates within the mitochondrial inter-membrane space [47] upon specific stimuli, including pro-apoptotic stresses [48] or growth factor stimulation [49], and oxidises cytochrome *c* to form H_2_O_2_ [50], which in turn regulates mitochondrial [51], and cellular functions [49, 52]. Accordingly, cells from p66Shc null mice or p66Shc-depleted by RNAi have reduced ROS levels [49]; however, p66Shc null mice show normal tumour incidence [53] and increased mutation rate [54]. Notably, p66Shc deletion is counter-selected when mice are maintained in harsh settings that mimic conditions in the wild (in an open field in the cold and in competition for food), indicating that the pro-oxidant function of p66Shc is essential for fitness under stressing natural conditions but redundant in a protected environment [55].

## The role of oxidative stress in cancer

Tumorigenesis is characterised by major alterations in energetic metabolism, O_2_ consumption and ROS accumulation [2] which result in a change in the balance between reduced/oxidised species (redox balance) [56]. Changes in the cellular redox balance affect proliferation, migration, and survival of cancer cells contributing to disease progression [57–59]. Activated oncogenes, such as Myc [60], Bcl-2 [61] or Ras [62], have been reported to affect redox balance. For example, the expression of the oncogenic form of Ras was found to boost [63–65] or to reduce [66] the level of glutathione, depending on the cell line. Furthermore, a reducing environment associates with different types of cancer [67–68]. This “reducing” environment results from the relative concentration of all the oxidant and reducing species (redox species) that exist in the metabolic network [69]. As a consequence, high levels of ROS endogenous scavengers or treatment with antioxidants increase oncogenic transformation [70] or tumour progression [71, 72], whereas increasing oxidation has even been proposed as a therapeutic strategy for cancer [73–76]. Finally, the antioxidant activity of chemicals is considered hazardous for novel classes of anticancer drugs [77, 78].

## Conclusion

Antioxidants affecting ROS levels and functions produce different outcomes since ROS have both deleterious and beneficial effects. Although ROS are a by-product of aerobic metabolism, several enzymatic systems have evolved to generate ROS on purpose. In cancer cells, ROS act as secondary messengers of oncogenic signalling pathways and can also induce cellular senescence and apoptosis. As a consequence, oxidative stress during cancer expansion and progression selects for clones with high antioxidant metabolism. Based on this, the failure of antioxidant treatments, as documented in several clinical trials, is not surprising. Antioxidant drugs are not sufficient to inhibit tumorigenesis or, even worst, they may accelerate it.
